# Glutathione Supplementation as an Adjunctive Therapy in COVID-19

**DOI:** 10.3390/antiox9100914

**Published:** 2020-09-25

**Authors:** Vika Guloyan, Buzand Oganesian, Nicole Baghdasaryan, Christopher Yeh, Manpreet Singh, Frederick Guilford, Yu-Sam Ting, Vishwanath Venketaraman

**Affiliations:** 1 College of Osteopathic Medicine of the Pacific, Western University of Health Sciences, Pomona, CA 91766-1854, USA; vika.guloyan@westernu.edu (V.G.); buzand.oganesian@westernu.edu (B.O.); nicole.baghdasaryan@westernu.edu (N.B.); christopher.yeh@westernu.edu (C.Y.); yusam.ting@westernu.edu (Y.-S.T.); 2 Department of Emergency Medicine, St Barnabas Hospital, Bronx, NY 10457, USA; preetysinghr@sbcglobal.net; 3 Your Energy Systems, Palo Alto, CA 94301, USA; drg@readisorb.com

**Keywords:** COVID-19, glutathione, cytokines, TNF-α, IL-6

## Abstract

Morbidity and mortality of coronavirus disease 2019 (COVID-19) are due in large part to severe cytokine storm and hypercoagulable state brought on by dysregulated host-inflammatory immune response, ultimately leading to multi-organ failure. Exacerbated oxidative stress caused by increased levels of interleukin (IL)-6 and tumor necrosis factor α (TNF-α) along with decreased levels of interferon α and interferon β (IFN-α, IFN-β) are mainly believed to drive the disease process. Based on the evidence attesting to the ability of glutathione (GSH) to inhibit viral replication and decrease levels of IL-6 in human immunodeficiency virus (HIV) and tuberculosis (TB) patients, as well as beneficial effects of GSH on other pulmonary diseases processes, we believe the use of liposomal GSH could be beneficial in COVID-19 patients. This review discusses the epidemiology, transmission, and clinical presentation of COVID-19 with a focus on its pathogenesis and the possible use of liposomal GSH as an adjunctive treatment to the current treatment modalities in COVID-19 patients.

## 1. Introduction

With approximately 11 million confirmed cases and over 525,000 deaths documented, the novel strain of coronavirus, which initially emerged at the end of 2019 in Hubei Province of the People’s Republic of China, has been found to precipitate clinical acute respiratory distress syndrome (ARDS) [1]. Coronaviridae are a large family of enveloped RNA viruses with virulent capacity observed across many species. Investigation of this novel emergent strain is ongoing and hence its pathological features have yet to be fully revealed. The first reports of the disease were reported as a cluster of unusual community acquired pneumonia cases concentrated in Hubei Province of the People’s Republic of China in December of 2019. On January 7th, 2020 the causative pathogen was identified as a novel coronavirus and provisionally named “2019-nCoVID [1]. One month later, the World Health Organization (WHO) declared the situation to be a public health emergency of international concern. The virus was then officially designated severe acute respiratory syndrome coronavirus 2 (SARS-CoV-2) for its apparent genetic and zoonotic similarity to SARS-CoV-1, formerly known as SARS-CoV, and Middle East respiratory syndrome coronavirus (MERS-CoV). The disease caused by SARS-CoV-2 is termed COVID-19.

The challenges of responding to this pandemic have been attributed to a myriad of factors including incomplete knowledge of SARS-CoV-2 pathophysiology, the mechanisms of action by which it causes harm, and the immune responses of individuals with comorbidities. Widespread shortages of personal protective equipment and testing systems have hamstrung organized response. Clinical trials are taking place assessing efficacy of the protease inhibitors lopinavir-ritonavir, RNA polymerase inhibitor Remdesivir, the antimalarial hydroxychloroquine, and IFN-1B as possible treatments in adjunct to supportive care [2]. The only long-term solution is seen in a form of vaccine, which is yet to be developed. The role of cytokine dysregulation in COVID-19 pathophysiology has been documented in multiple studies. This provides the scientific community with the foundation to propose possible mechanisms of pathogenesis and develop treatment modalities to limit morbidity and mortality.

## 2. SARS-CoV-2 Microbiology

Genetic sequencing studies have grouped SARS-CoV-2 along with SARS-CoV-1 and MERS-CoV into the genus Betacoronavirus in the family Coronaviridae. SARS-CoV-2 is a spherical, pleomorphic enveloped virus containing a 30 kbp positive-sense single-stranded RNA genome. The family Coronaviridae possess the largest genomes of all known RNA viruses [3].

The main structural components of SARS-CoV-2 are the protein spike (S), membrane (M), envelope (E), and nucleocapsid (N). The M glycoprotein is the most abundant structural protein and is responsible for the intracellular host-assembly of viral particles [3]. The S protein is a club-shaped glycoprotein found on both SARS-CoV-1 and SARS-CoV-2 that binds to angiotensin-converting enzyme 2 (ACE-2) on potential host cells [4]. Viral entry is demonstrated to require the activity of the cysteine proteases cathepsin B and cathepsin L (CatB/L) as well as the serine protease TMPRSS2 for S protein priming. Aloxistatin and camostat mesylate, which inhibit CatB/L and TMPRSS2, respectively, are shown to prevent entry of SARS-CoV-2 into human cells when used in combination [5]. These medications are being investigated as a potential treatment modality for COVID-19 [5]. Incubation of SARS-CoV-2 in the presence of tunicamycin results in non-infectious virus particles with absent S protein [4,6]. The S protein is also the inducer of neutralizing antibodies in the host, which makes the S protein an ideal target for the vaccines [4,6].

A study conducted on SARS-CoV-1, the strain of Coronaviridae attributed to the 2002–2004 SARS outbreak, found that intracellular response to the virus is mediated by RNA-activated protein kinase (PKR) in human cells expressing ACE-2. The molecular mechanism of this response involves phosphorylation of the translational initiator eukaryotic initiation factor 2-α) at Ser51 residues. Phosphorylation of eIF2-α results in global inhibition of mRNA translation. However, this inhibition of eIF2-α was not found to significantly decrease viral replication. It has been proposed that this mechanism is a viral adaptation to limit host-immune response by inhibition of translation of host-response proteins [7].

## 3. Transmission

Early data suggest that horseshoe bats and pangolins are likely mammalian reservoirs for SARS-CoV-2. However, the intermediate host through which direct interspecies transmission to humans occurred remains uncertain. Transmission of SARS-CoV-2 between human hosts is mediated by aerosolized respiratory droplets expectorated by an infected host. Transmission can also occur during aerosol-generating procedures including endotracheal intubation and administration of nebulizer therapy. Human-to-human transmission by asymptomatic carriers makes containment of SARS-CoV-2 difficult, as 40–50% of cases are caused by transmission from asymptomatic carriers [8,9]. The R_o_ of SARS-CoV-2 is estimated to be 3.28 in a recent review article, meaning that each infected host is expected to transmit the infection to approximately three healthy individuals. Data estimates for R_o_ vary across the literature and will likely continue to evolve as the pandemic progresses [10].

## 4. COVID-19 Epidemiology

According to CDC data, the age ranges of 18–44 and 45–64 comprise the majority COVID-19 cases, with 0–17-year-olds being affected the least [2]. In addition, males are affected more than females. A case series from China which surveyed 1099 hospitalized patients with laboratory confirmed SARS-CoV-2 infection, revealed that 41.9% of patients were female and 58.1% were male [11]. Significant risk factors for developing severe COVID-19 related illness include age of 65 or greater and the presence of comorbidities such as hypertension, chronic obstructive pulmonary disease (COPD), obesity, and diabetes [2]. According to a meta-analysis which assessed data from 1558 patients, increased risk for severe COVID-19 related illness was observed in patients with hypertension (OR = 2.29, *p* < 0.001), diabetes (OR = 2.47, *p* < 0.001), COPD (OR = 5.97, *p* < 0.001), cardiovascular disease (OR = 2.93, *p* < 0.001), and cerebrovascular disease (OR = 3.89, *p* = 0.002) [12]. Young patients without significant medical history may also require ICU care or die due to COVID-19. The reason behind this phenomenon is thought to be an excessive host-inflammatory immune response precipitating as cytokine storm, septic shock, and multiorgan failure. Currently, the case fatality rate of COVID-19 is estimated to be at 2% [2]. The accuracy of this data, however, is difficult to assess due to failure to conduct testing on those with mild or asymptomatic disease. Therefore, case fatality rates are likely lower than current estimates. Autopsy reports have found that the virus had infected the heart in 41 percent of patients age 78 to 89 years old [13]. Additionally, it is reported that three-quarters of recovered COVID-19 patients were left with structural changes to their hearts, even two months later [14].

## 5. Clinical Presentation and Diagnosis

A study of 191 patients with confirmed SARS-CoV-2 showed the median incubation period to be 5.1 days from time of exposure, with 97.5% developing symptoms after 11.5 days [15]. The most common symptoms of COVID-19 are cough, dyspnea, and fever. Diarrhea is also reported in several cases. A case series of 1099 hospitalized patients with laboratory confirmed SARS-CoV-2 infection in China demonstrated that 43.8% presented with fever on admission, while 88.7% developed fever during hospitalization. A total of 67.8% of patients presented with cough [11]. Another case series of 393 patients in New York City showed dyspnea was a presenting symptom in 56.5% of cases [16].

Interestingly, early manifestations may often include hyposmia and hypogeusia. Expression of angiotensin-converting enzyme 2 (ACE2) has recently been found to be high in the oropharynx and tongue. Thus, ACE2 receptor binding by SARS-CoV-2 in COVID-19 may explain the loss of smell and taste (anosmia and dysgeusia) observed in patients in the early stages of COVID-19. It is intriguing to speculate that the binding of the S protein of SARS-CoV-2 to ACE2 stimulates an oxidation reaction similar to what we have seen with the binding of other viruses like influenza [17]. At the present time, it is unclear whether this olfactory dysfunction results from viral-induced olfactory nerve damage or local inflammation of the nasal cavity or both [18].

Upon clinical suspicion for COVID-19 based on history and low oxygen saturation, most emergency physicians will obtain initial blood work to analyze inflammatory markers to make presumptive diagnosis while waiting for confirmatory SARS-CoV-2 results. In the early stages, COVID-19 is most often confirmed by reverse transcriptase polymerase chain reaction (RT-PCR) of nasopharyngeal or oropharyngeal swabs. This assay may take up to 48 h to perform, hence initial blood work must be used in the interim to monitor disease progression. These studies included complete blood count with differential, chemistry panel including liver function tests, troponin and brain natriuretic peptide, procalcitonin, ferritin, erythrocyte sedimentation rate, C-reactive protein (CRP), lactate dehydrogenase (LDH), D-dimer, creatine phosphokinase (CPK), interleuking-6 (IL-6), blood/sputum cultures, and urine antigen for legionella pneumococcus. Additional studies include chest radiograph and point of care ultrasound to rule out pneumothorax, pleural effusion, pericardial effusion, heart failure, and an electrocardiogram for patient’s baseline line QTc for determination of which medication to administer. Initial workup may vary by institutional policy. Mardani et al. showed that patients with positive RT-PCR for SARS-CoV-2 had significantly increased neutrophil count, CPK, LDH, liver function enzymes, and erythrocyte sedimentation rate. These findings are accompanied by leukopenia [19,20]. Fan et al. evaluated hematological studies of COVID-19 infected patients between intensive care unit (ICU) and non-ICU patients demonstrated that lymphopenia and elevated LDH are associated with increased rate of ICU admission [21]. Multiple studies show that elevated expression of IL-6 can be used to predict the severity of COVID-19, with increased need for ICU care and progression to ARDS [22,23,24]. Severe disease and mortality appear to be associated with elevated LDH, procalcitonin, ferritin, and IL-6 [25]. Another study showed that serum ferritin level was associated with the progression to ARDS [24]. Finally a systematic review on the role of biomarkers in COVID-19, which looked at 26 different articles, concluded that IL-6 CRP, LDH, D-dimer, troponin, blood urea nitrogen and creatinine were elevated while leukocyte count is decreased in patients with severe SARS-CoV-2 infection [26].

Alternatively, another avenue of testing is serology of serum for SARS-CoV-2 IgA, IgM, or IgG antibodies. While RT-PCR is relatively rapid and better-suited to testing in an acute setting, serology screening shows promise as a useful method for confirming an ongoing or prior infection [27].

The degree to which the presence of serum anti-SARS-CoV-2 immunoglobulins confers secondary immunity to COVID-19 has yet to be determined in humans. Therefore, the risk for reinfection by the virus after initial recovery remains a concern for healthcare systems globally. In a promising study conducted in rhesus macaques, individuals were challenged with infectious doses of intratracheal SARS-CoV-2 and monitored. At 28 days post-infection, monkeys were re-challenged with an identical dose of intratracheal SARS-CoV-2. Re-infected individuals exhibited decreased nasopharyngeal viral load, increased proportion of CD4+ T cells, and increased serum concentrations of neutralizing antibodies. Interestingly, re-infected monkeys exhibited a transient fever which was not observed in initial inoculation [28].

## 6. Pathogenesis of COVID-19: An Evolving Picture

**Figure 1:**
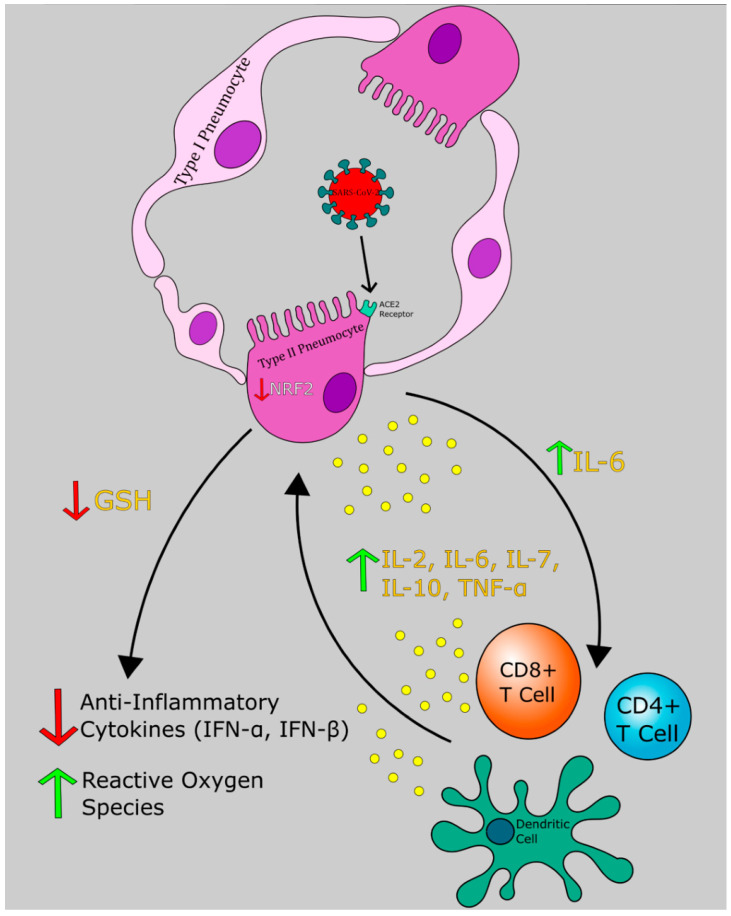
Severe acute respiratory syndrome coronavirus 2 (SARS-CoV-2) induced cytokine storm and redox imbalance. SARS-CoV-2 binds to the angiotensin-converting enzyme 2 (ACE2) receptor and induces down regulation of nuclear factor erythroid 2-related factor 2 (NRF2), which leads to inhibition of glutathione (GSH) release. This results in elevated inflammatory cytokines, elevated reactive oxygen species (ROS), and recruitment of immune cells. Arrows indicate secretion of cytokines and their downstream effectors.

The highly variable clinical course of COVID-19 complicates diagnosis and treatment. Of infected individuals, many presented with mild respiratory symptoms and an unknowable number have no symptoms at all. As more serology assays are conducted confirming prior infection, evidence of many asymptomatic infections is emerging. Certain patients infected with SARS-CoV-2 tend to develop mild or asymptomatic disease while others progress to severe pneumonia and ARDS. We hypothesize that the variability in the illness course stems from the imbalance between proinflammatory and anti-inflammatory mediators in lung parenchyma (Figure 1). The distinct pathogenesis of COVID-19 can be divided into 3 phases: initial infection of the respiratory tract, acute inflammation, and either resolution or severe disease.

### 6.1. Phase I: Respiratory Tract Infection

Initial infection begins upon inhalation of aerosolized SARS-CoV-2 virions. SARS-CoV-2 enters the nasal mucosa and causes swelling and inflammation. The ciliated epithelial cells and mucus-producing goblet cells of the respiratory tract expel the pathogen via the mucociliary escalator. Patients with a history of diabetes, cardiovascular disease or tobacco use have an impaired ability to evacuate the pathogen via this innate mechanism. This places them at a higher risk of the virus colonizing the lower respiratory tract [29]. Infected persons are typically asymptomatic, due to the lack of colonization of the lower respiratory system. Host defenses in the respiratory epithelial lining such as glutathione (GSH)), beta-defensins, and immunoglobulin A (IgA), up-regulate innate and adaptive immune responses against infections and may help clear infection before it progresses. During this initial phase, infected patients are most likely to have a nasopharyngeal swab test positive for SARS-CoV-2 via RT-PCR [30].

If the virus reaches the lower respiratory tract, it will bind to ACE2 on type II pneumocytes using its S protein. Then, SARS-CoV-2 enters the host cell, releasing its single-stranded RNA (ssRNA) genome, and uses host machinery to replicate. Type II pneumocytes are responsible for surfactant production and have regenerative capacity for type I pneumocytes, which participate in respiratory gas exchange. As a result, damage to type II pneumocytes leads to decreased surfactant and diminished type I pneumocyte pool, leading to impaired gas exchange, decreased compliance, pulmonary edema and pneumonia (Figure 1) [31].

ACE2 receptors are also expressed in intestinal epithelial cells, allowing SARS-CoV-2 to infect enterocytes. This explains initial GI symptoms such as diarrhea in a subset of COVID-19 patients [11].

### 6.2. Phase II: Acute Inflammation

**Figure 2:**
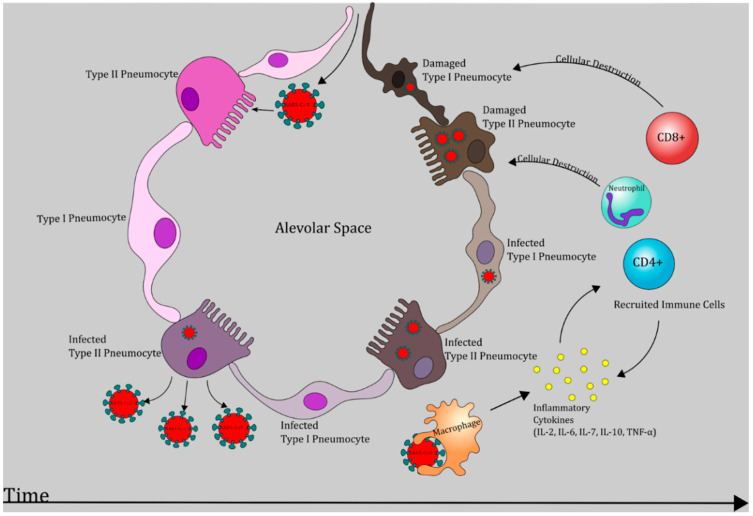
SARS-CoV-2 induced pathogenesis in the lungs. Chronological representation of SARS-CoV-2 progression leading to necrosis of type I and type II pneumocytes which may manifest as respiratory dysfunction and acute respiratory distress syndrome (ARDS) [6,27].

Local immune response in the lung parenchyma is mediated by alveolar macrophages, dendritic cells and type II pneumocytes. Macrophages and type II pneumocytes secrete IL-8, which acts a powerful neutrophil chemo-attractant [32]. Acute inflammation ensues with migration of a large number of neutrophils and monocytes to the alveoli [33]. Subsequent degranulation releases acute phase reactants such as IL-6 and tumor necrosis factor α (TNF-α). Phagocytic leukocytes utilize reduced nicotinamde adenine dinucleotide phosphate (NADPH) as a cofactor for NADPH oxidase (NOX) to catalyze the generation of the reactive oxygen species (ROS), hydrogen peroxide, and hypochlorite [34]. Production of these highly reactive substances coupled with inundation by pro-inflammatory cytokines are the molecular basis of severe parenchymal injury due to COVID-19 (Figure 1 and Figure 2). A study of laboratory measurements of ROS in four patients with confirmed COVID-19 has revealed that lower levels of ROS are correlated with shorter course of illness [35].

IFN-α and IFN-β, otherwise known as type I interferons, have been demonstrated to play a role in the innate component of the immune response to viral infection (Figure 1). Several strains of flavivirus possess adaptations which antagonize IFN-α/β signaling by inhibiting the JAK/STAT pathway [36,37]. An experiment performed in human embryonic kidney (HEK293) cells which transgenically express SARS-CoV-2 structural proteins, identified that N protein, ORF6, and ORF8 genes antagonize activity of IFN-β and NF-κβ [38]. Additionally, in vitro incubation of Vero cells with recombinant human IFN-α resulted in markedly decrease in viral titers following challenge with SARS-CoV-2 when compared to mock-infected controls [39].

Further destruction of intracellular pathogens is carried out by cytotoxic T lymphocytes (CTLs). Antigens displayed on antigen presenting cells along with major histocompatibilty complex (MHC)I are recognized by CTLs that express the appropriate T cell receptor (Figure 1 and Figure 2). Subsequent degranulation of CTLs releases cytotoxic perforin and granzyme-B. Perforin increases permeability of virus-infected cell membranes and allows granzymes to proteolytically activate intracellular caspases which ultimately initiate apoptosis [40]. In response to antigen presentation, Th1 and Th2 subtypes release IFN-γ, IL-2, IL-4, IL-6, granulocyte-macrophage colony stimulating factor (GM-CSF), and IL-10. IL-2 stimulates differentiation of both helper and cytotoxic T cells in the bone marrow, while IL-4 promotes B cell growth and antibody class switching [41]. IFN-γ enhances macrophage and monocyte driven phagocytosis and enhances virus-infected cell destruction by NK cells [42]. GM-CSF is a glycoprotein which promotes differentiation of neutrophils and CD-14/CD-16 monocytes in the bone marrow. This subset of monocytes releases high levels of IL-6 and is not found in healthy individuals [43]. IL-10 is an anti-inflammatory cytokine largely providing inhibitory feedback to the immune response to excessive host tissue destruction by neutrophils, macrophages, NK, and T cells [44].

At the center of the inflammatory positive feedback loop are TNF-α and IL-6. Macrophages, neutrophils and activated lymphocytes release TNF-α and IL-6. IL-6 causes fever and production of more acute phase proteins. TNF-α causes further recruitment of leukocytes and plays a role in development of septic shock and disseminated intravascular coagulopathy [45].

Gomez-Pastora et al. reviewed retrospective studies which measured serum ferritin and IL-6 levels in patients with severe and non-severe COVID-19 disease and tried to decipher whether increased ferritin and IL-6 levels were markers of severe disease or disease progression. Their conclusion was that hyperferritinemia, defined as ferritin level > 400, was noted in most patients with severe COVID-19 disease on admission with an average ferritin level of 800, which was about 1.5–5.3 times more than in patients with less severe disease. In addition, non-survivors had ferritin levels around 1400 μg/L, 3 and 4 times higher than that observed in survivors [46]. In addition, Liu et al. showed that ferritin and IL-6 can be used to monitor disease progression and severity of cytokine storm, as recovering patients were observed to have down trending ferritin and IL-6 levels [23]. Hyperferritinemia and increased synthesis of hepcidin is observed in inflammatory states and is believed to be a result of cytokine storm and high levels of IL-6. The significance of this is that ferritin and hepcidin sequester iron intracellularly, resulting in formation of ROS, reactive nitrogen species (RNS), and reactive sulfur species and may contribute to tissue damage in the lungs and other involved organs [47,48]. Iron also interacts with clotting factors in the coagulation cascade and leads to a hypercoagulable state according to Jankun et al. [49]. Furthermore, increased intracellular iron can contribute to a novel mechanism of cell death called ferroptosis [50].

### 6.3. Phase II: Resolution or Severe Illness and Hypercoagulable State

#### 6.3.1. Resolution

As stated by the CDC, full recovery from COVID-19 is defined as resolution of fever in the absence of fever-reducing medical therapy along with resolution of respiratory symptoms [12]. Adequate immune response preventing severe illness in patients can be attributed to antiviral immune factors such as T-regulatory cells, antibody production by B cells, adequate IFN-α and IFN-β action. T-regulatory cells suppress further immune cell stimulation by suppressing CD4 and CD8 T cell effector function. They also produce IL-10 and transforming growth factor β (TGF-β), which together inhibit macrophages and dendritic cells, decrease MHC II expression, and decrease Th1 cytokine release. The action of these anti-inflammatory mediators, together with cell mediated immunity carried out by NK cells and CTLs eradicates virus from host cells and leads to resolution of the infection (Figure 1) [51].

#### 6.3.2. Severe Illness

Inpatient and outpatient management of COVID-19 is complicated by its widely variable clinical course. Deterioration of respiratory function can be sudden and rapid. In some patients, the immune response leads to viral destruction and downregulation of inflammation, while others generate a severe proinflammatory hypercytokinemia, multisystem inflammation, and shock, with some patients developing respiratory failure requiring mechanical ventilation [52] (Figure 1). 

## 7. Interleukin-6 and ARDS

Progression to ARDS in a subset of COVID-19 patients can be partially attributed to dysregulation of host cytokine production precipitating a positive feedback cycle of inflammatory insult and parenchymal injury. Subsequent increased capillary membrane permeability and fluid buildup in lungs and explains the development of ARDS requiring the need for mechanical ventilation. This is supported by studies which have shown increased acute phase reactant levels in COVID-19 patients. A study in Munich conducted a statistical assessment of baseline laboratory values in 40 patients with RT-PCR confirmed SARS-CoV-2 infection. Results demonstrated that elevated plasma concentrations of IL-6 are strongly associated with the need for mechanical ventilation (*p* = 1.2 × 10^–5^) and respiratory failure (*p* = 1.7 × 10^–8^). A total of 13 out of the 40 patients who required mechanical ventilation did not at baseline have any differences in comorbidities, radiological findings or quick sequential organ failure assessment (qSofa) scores had significantly elevated IL-6 levels compared to the 27 patients who did not need mechanical ventilation. The statistically optimal predictive threshold for plasma IL-6 concentration was determined to be 80 pg/mL [53]. 

In order to determine the pathogenesis of this novel disease, it may be prudent to analyze data on SARS-CoV-1, with which SARS-CoV-2 shares genetic similarity. The initial phases of SARS-CoV-1 replication involves translocation of the N protein to the host nucleolus; N protein may co-localize to both the cytoplasm and nucleolus. N protein is multifunctional and facilitates packaging of the viral genome [54]. SARS-CoV-1 N protein activates the IL-6 promoter and subsequent IL-6 transcription as demonstrated in A549 human lung cells [55]. It has been shown that SARS-CoV-1 N protein interacts with the host transcriptional factor NF-κB in a dose-dependent manner to regulate IL-6 expression [55,56]. A 2011 study showed that IL-6 induced a dose-dependent decrease in intracellular GSH levels in a number of human cell lines, including lung cells [57]. Decreased GSH has been shown to be associated with an increase in IL-6. The likelihood that IL-6 and GSH share an inverse relationship is heightened by the observation that the administration of exogenous GSH in GSH-depleted, HIV-positive patients results in decreased levels of IL-6 [58,59]. It appears that infection with COVID-19 can stimulate a positive feedback cycle of increased IL-6 and decreased GSH that may explain the cytokine storm that can accompany this infection [56]. Studies of individuals with HIV stabilized on antiretroviral therapy have shown that this persistent decrease in GSH is accompanied by an elevation of IL-6, IL-10, and TGF-β. The relationship with GSH is demonstrated by the observation that these cytokines can be returned toward baseline values by the use of exogenous GSH in the form of liposomal GSH [58,59].

A study conducted in mice demonstrated that IL-6 is markedly elevated in individuals after hypercytokinemia was induced by peroxidized phospholipids and intrapulmonary acid. IL-6 -/- mutant mice exposed to the same cytokine storm-inducing stimuli exhibited improved lung function, decreased edema, decreased lung pathology on hematoxylin/eosin stain as compared to wild-type specimens. This process appears to be mediated by a TLR4 dependent mechanism [60].

The IL-6 receptor antagonist, tocilizumab, is an immunosuppressive monoclonal antibody currently under investigation as a potential intervention for COVID-19. A retrospective cohort study was conducted in 544 adult patients with severe COVID-19 related illness. The authors found a statistically significant decrease in mortality in patients receiving tocilizumab with standard care (7%) as compared to those receiving standard care alone (20%) with a *p*-value of <0.0001 [61].

## 8. Septic Shock and Hypercoagulability in COVID-19

Severe infection with SARS-CoV-2 results in hypercoagulable state. Up to 50% of patients with mild or severe COVID-19 develop associated coagulopathy with evidence of venous thromboembolism (VTE) and pulmonary embolism (PE) [62]. A single-center cohort study measured the incidence of VTE in a cohort of 198 hospitalized patients who showed evidence of thrombotic complications including D-dimer elevation, symptomatic PE, and VTE associated mortality in the ICU [63]. 

Hypercoagulability is a prothrombotic state which occurs in the setting of endothelial tissue damage. The fibrin degradation fragment, D-dimer, is a useful clinical indicator of embolic turnover in SARS-CoV-2 infection. A cohort study was performed on 279 laboratory-confirmed COVID-19 patients who were stratified to groups with mild-moderate disease (*n* = 136), improved disease (*n* = 23), and severe disease or death (*n* = 120). Serum D-dimer concentrations were monitored for ten days after admission. The authors concluded that serum D-dimer concentrations were markedly elevated, and continued to increase through hospital stay, in subjects with severe disease when compared to those with mild or improved symptoms. The CDC has clearly established the correlation of elevated D-dimers with poorer outcomes in patients with COVID-19 [64].

During acute inflammation, TNF-α is produced by macrophages which leads to a cascade of events that results in cell death via necrosis and apoptosis. TNF-α induces release of the substance tissue factor which activates the extrinsic pathway of the coagulation cascade. The end result of this cascade is the production of cross-linked fibrin clots. The dissolution of venous thromboemboli is mediated by a process known as fibrinolysis. Tissue-plasminogen activator (TPA) converts plasminogen to plasmin, which then catalyzes fibrinolysis. Under normal conditions, plasminogen activator inhibitor-1 (PAI-1) is tonically inhibited by protein C. In excess of TNF-α, protein C levels are reduced. In the absence of protein C mediated inhibition of PAI-1, less plasminogen is generated resulting in a prothrombotic state. This ultimately leads to persistence of venous thromboembolism [65]. 

## 9. Endogenous Glutathione Synthesis and Regulation the Role of Redox Cycle in Reducing Oxidative Stress Redox Cycle, Antioxidant Clearance, and Associated Isoenzymes Involved

GSH is a biological antioxidant that can be found in the mitochondria of all mammalian tissues and is highly involved in mitigation of oxidative stress. It is a pleiotropic tripeptide composed of glycine, cysteine, and glutamate [66]. Free GSH synthesis occurs intracellularly in two steps. The first step in GSH synthesis uses glutamine and cysteine as substrates and is catalyzed by glutamine-cysteine ligase (GCL) producing γ-glutamyl cysteine. The second step adds glycine to γ-glutamyl cysteine and is catalyzed by glutathione synthase (GS). Reduction of glutathione disulfide (GSSG) by GSH reductase using NADPH as a cofactor also results in formation of 2 GSH molecules [67]. The active thiol group of the cysteine residue participates in antioxidant functioning by providing reducing equivalents for the eradication of ROS and (RNS) or indirectly via GSH-dependent peroxidase catalyzed reactions 66. Therefore, the role of GSH in the human cell is to prevent oxidative stress induced cellular damage. There will be an increase in GSSG in cells that are undergoing oxidative stress [66]. The GSH redox cycle is one of the major enzymatic cell defense systems that protects cells against damage from peroxides such as hydrogen peroxide and lipid hydroperoxide. A key enzymatic step in antioxidant clearance via the GSH redox cycle is the conversion of hydrogen peroxide to water (H2O), which is catalyzed by the enzyme glutathione peroxidase (GPx). This reduction of hydrogen peroxide by GPx occurs at the expense of oxidizing GSH to its disulfide (GSSG) form. GSSG is subsequently reduced back to GSH in the body via the enzyme glutathione reductase by utilizing the reducing agent NADPH. It has been shown that intracellular glutathione peroxidase comes in both the classical (cGPx) and phospholipid hydroperoxide (PHGPx) forms. Both cGPx and PHGPx belong to a family of proteins known as the selenoproteins, whose active sites consist of the amino acid selenocysteine. Although these proteins share a selenocysteine group, previous studies have shown distinct features in the substrate specificity of these enzymes, eluding to the idea of both shared and independent roles of these antioxidant enzymes [66].

As illustrated by Maiorino et al., there are four total isoenzymes for the GPx family that are currently recognized. Their shared features include a catalytic triad composed of a selenocysteine active site, as well as conserved tryptophan and glutamine residues [68]. These isoenzymes include the cGPx, GPx-GI, pGPx, and PHDPx proteins, or GPx1-GPx4, respectively. GPx1 is a cytosolic protein that has been well studied. It is shown by Mills et al. to play a role in preventing red blood cell hemolysis and the reduction of hydrogen peroxide, amongst other peroxides [69]. GPx2 is also an intracellular glutathione peroxidase with specific activity in the gastrointestinal tract. GPx3 is active in the plasma and is distinct due to its antioxidant role being extracellular [66].

Unlike the tetrameric GPx1-GPx3, the GPx4 protein is a monomer which can not only reduce hydrogen peroxide, but also distinctly react with lipid and fatty acid hydroperoxides including cholesterol derivatives, amongst many other substrates. PHGPx’s role in many tissues has been described, including protection against oxidative stress against spermatozoa in the testes, as well as its neuroprotective properties illustrated by the link between GPx4 deficiency and Alzheimer’s and Parkinson’s dementia [66].

## 10. Glutathione and Tuberculosis

It has been reported that GSH levels are significantly diminished in peripheral blood mononuclear cells and red blood cells of individuals with pulmonary TB. In patients with TB, GSH deficiency is associated with elevated ROS, production of pro-inflammatory cytokines and *Mycobacterium tuberculosis* (*M. tb*) persistence. The major innate immune cells that are responsible for combating *M. tb* infections are macrophages, which rely on antimicrobial molecules such as RNS to inhibit the growth of *M. tb*. RNS such as nitric oxide are short-lived molecules that either are rapidly detoxified in the cytosol or react with various cellular components interfering with their function. GSH has been shown to act as a shuttle for nitric oxide by forming S-nitrosoglutathione (GSNO). Once formed, GSNO can release NO and cause intracellular death of the pathogen [70,71,72,73]. Aside from its role as a carrier molecule for NO, GSH limited intracellular growth of *M. bovis* and *M. tb* in macrophages with inducible nitric oxide synthase knockout in mice. This concludes that GSH has direct antimycobacterial activity distinct from its role as a NO carrier [70].

Natural killer (NK) cells have an important role against *M. tb* infection and their activity can become critically impaired in patients with low levels of GSH [71]. Studies have shown that NK cells that are given the GSH precursor N-acetyl cysteine (NAC) as well as IL-2 and IL-12 display a recovery of cytolytic activity and decrease *M. tb* viability in infected cells. When given NAC, IL-2, and IL-12 there was an increase in the cell surface expression of FasL and CD40L on the NK cells which play an important role in signaling apoptosis of cells infected with *M. tb* [72].

CD4+ T cells are lymphocytes that play an important role in mounting a proper and robust immune response against a pathogen, especially TB. Although T-helper cells are not directly involved in the killing, they assist and activate other adaptive and innate immune cells by releasing cytokines and thus killing the pathogen. For example, in the presence of IL-12 and IFN-γ, T helper cells can differentiate into Th1 cells which play an important role in the host defense against intracellular pathogens [72]. GSH has an important role in the modulation of cytokine expression. In vitro treatment of whole blood with NAC has resulted in an increase in IFN-γ production, thus increasing the Th1 cellular response to *M. tb*, thus controlling the growth of *M. tb* [71]. 

## 11. Glutathione and Viral Infections

According to the WHO, there were 37.9 million people living with HIV in the globe by the end of 2019 [73]. HIV infection is associated with susceptibility to infections, such as TB, secondary to immunocompromise that occurs following depletion of the CD4+ cells [74]. Our laboratory has shown that HIV infected individuals have a deficiency in intracellular GSH in RBCs, T cells, NK cells, and monocytes [53,58]. A double-blind placebo controlled clinical trial conducted by our laboratory has demonstrated that the GSH that was present in macrophages in patients with HIV was in the oxidized state (GSSG) which lacks the antioxidant properties of reduced GSH [58]. The decreased levels of reduced GSH correlated with an increase in the growth of *M. tb* in macrophages of patients infected with HIV. There also was a decrease in the expression of genes coding for enzymes responsible for the synthesis of GSH. Our labs showed that supplementing macrophages from HIV positive patients with L-GSH led to a decrease in ROS production and improvement of macrophages ability to kill *M.tb* [74].

Additionally, our published findings have shown a reduction in levels of immune stimulatory cytokines (IL-2, IL-12, and IFN-γ) and an increase in immune suppressing cytokines (TGF-β, IL-6, IL-10) among HIV infected patients [59]. TGF-β has been shown to downregulate activity of Glutamine Cysteine ligase, a rate-limiting step enzyme involved in the synthesis of GSH [75]. Oral liposomal GSH supplementation for 3 months resulted in a restoration of the immune stimulatory cytokines and a decrease in the levels of immunosuppressive cytokines, thus improving immune function in patients with HIV [59]. These data demonstrate that the chronic inflammation due to HIV infection has led to a depletion of immune cells function due to depletion of reduced GSH production and as a result increases susceptibility to infections especially *M. tb* [74]. GSH depletion is a direct consequence of viral infection [74]. During viral infections, intracellular GSH depletion occurs through multiple mechanisms and is necessary for viral replication. It has become apparent that an oxidized state favors maturation of viral hemagglutinin (HA) in the endoplasmic reticulum via a protein disulfide isomerase (PDI)-mediated mechanism. Absence of these binding proteins inhibits viral replication [76,77].

Increased oxidative stress has been reported in several different viral infections both in vitro; with herpes simplex type 1, HIV, and Sendai virus; and in vivo, with influenza [78,79,80,81,82]. These findings have resulted in studies using Sendai virus, also known as parainfluenza type 1, as a model for mammalian respiratory infection [80]. Several studies have been conducted in Madin–Darby canine kidney (MDCK) cells which are permissive to viral replication [83]. MDCK cells infected with Sendai virus showed depletion of GSH over two time periods. GSH concentration began to decline within minutes of infection and reached initial nadir at 20 min post-infection; an additional loss occurred gradually over the subsequent 24 h as viral replication was completed. It appeared that reduced GSH was leaked from the cell during viral infection [83]. GSH depletion at early stages of viral infection was also observed during the infection of VERO cells with clinically isolated herpes simplex type 1 virus, indicating a more general relationship between oxidative stress and viral replication [78]. The study on Sendai in 1997 showed that adsorption of Sendai virus to the cell wall caused a loss of GSH, which affected the Na^+^/H^+^ antiporter leading to a lower intracellular pH. It was known that acidic conditions favor many viral infections by accelerating the fusion process of virus to cell membrane, thus enhancing viral replication [84]. Later in the viral infection, GSH decrease appeared to be due to the preferential incorporation of cysteine residues into viral proteins, while the formation of mixed disulfides between GSH and cellular proteins was also observed [83]. It was shown in 1976 in Sendai virus, the proteins used to bind and subsequently fuse the virus to the host cell are glycoproteins that present as oligomers connected with Inter-peptide disulfide bonds. This disulfide bond is easily cleaved by materials that produce a reducing environment such as reduced GSH. Splitting these disulfide bonds is accompanied by loss of biologic activity of the binding protein and can prevent viral replication [76].

It was subsequently shown that many viruses—particularly enveloped species such as Coronaviridae, Poxviridae, and Paramyxoviridae—bind at sites that allow the virus to be internalized by endocytosis [85]. The endosome is acidic and the level of acidity continues to increase. This environment allows protonation of the binding glycoprotein and the action of enzymes to activate the binding protein to fuse the virus membrane to the surrounding membrane and release virus RNA into the cytoplasm [86]. Release of RNA viral material occurs with coronavirus fusion to cell membranes [87].

Thus, intravesicular acidic pH facilitates the viral-cell fusion process [88,89]. Fusion at mild acidic pH activation is common to highly pathogenic avian influenza virus (HPAIV) as this allows rapid fusion in contrast to low pathogenic avian influenza virus (LPAIV) and regulates high virulence in chickens [90]. The importance of pH for fusion and the continuation of the viral infection is illustrated in studies which use methods to alkalinize the endosomes. It has been shown that avian infectious bronchitis virus, a coronavirus, requires a low pH to accomplish coronavirus-cell fusion [91]. 

The antimalarial drug chloroquine, known since 1934, is able to increase the pH of cellular compartments. Chloroquine/hydroxychloroquine is concentrated within acidic organelles such as endosomes, Golgi vesicles, and lysosomes, where the pH is low. The effect of the weak base is to disrupt several enzymes which require low pH to allow the replication of several viruses, including members of the flaviviruses, retroviruses, and coronaviruses [88]. Despite in vitro suggestion of antiviral benefit, chloroquine/hydroxychloroquine have not shown benefit in human trials in HIV or Dengue [92]. The results of the first clinical studies evaluating the effect of hydroxychloroquine do not support any efficacy of this drug in patients with COVID-19, due to major methodological weaknesses. Yet, these preliminary studies have aroused enough media interest to stimulate a large number of studies world-wide. It has been shown subsequently that chloroquine increased viral production when the drug was applied to cells after viral adsorption [93]. Serious adverse drug reactions have been reported in patients with COVID-19 receiving hydroxychloroquine, justifying limitation of its prescription and to perform suitable cardiac and therapeutic drug monitoring [94].

Influenza viruses induce oxidative stress mediated by excess of reactive oxygen species (ROS) and a decrease of reduced GSH the main intracellular antioxidant, and that such conditions favor viral replication [95,96,97,98]. The production of ROS is mediated by the activity of the NOX family, which consists of seven members: NOX1 to NOX5 and the two dual oxidases, Duox1 and Duox2, expressed in most cell types [99]. While NOX2 plays a role in the killing of bacteria and fungi, it enhances the pathology caused by viruses of low and high pathogenicity, including influenza A viruses [100]. NOX4 is up-regulated following viral infection in lung epithelial cells, and it is responsible for the ROS generation that promotes the nuclear export of viral ribonucleoprotein favoring viral replication [96].

It has been shown in influenza virus infection that GSH depletion is pivotal for the folding and maturation of the viral glycoprotein haemagglutinin (HA) and therefore for viral replication [101]. This has led to speculation that viruses manipulate the function of NRF2 [102]. Not all viruses increase oxidation using NRF2 dysfunction. For example, a significant reduction of hepatic, plasmatic, and lymphocytic GSH levels were noted in patients chronically infected by hepatitis C virus (HCV), particularly with the 1b genotype. The mechanism appears to involve an increase in oxidation by means other than the degradation of NRF2. Increased oxidative stress in hepatitis C may be explained by chronic inflammation, and the continued generation of ROS/ RNS by NADPH oxidase 2 (NOX2) of Kupffer cells and polymorphonuclear cells in the liver [103]. NS3 protein of HCV has been found to activate NOX2 protein of phagocytes and to trigger apoptosis and dysfunction of T cells, natural killer cells, and natural killer T cells [104]. It is speculated that HCV, by damaging liver, may promote systemic oxidative stress in part by disruption of GSH export. Therefore, it may be hypothesized that HCV produces oxidative stress through multiple mechanisms that include chronic inflammation, iron overload, and liver injury [103]. A study evaluated the NRF2 expression during acute and chronic HCV infection phases, showing that the protein was downregulated during early phases of infection, while it was more expressed during the chronic phase [105]. 

Viruses possess a variety of adaptive mechanisms to deplete GSH in host cells. Respiratory Syncytial Virus (RSV) was shown to use NOX2 as an essential regulator of RSV-induced NF-κB activation. RSV infection induced a persistent activation of NF-κB, which likely led to excessive NF-κB-mediated inflammatory gene expression [106]. A later study found that RSV infection down-regulates NRF2 expression in airway epithelial cells and a decrease in the expression of airway antioxidant enzymes led to additional oxidative stress. NRF2 mRNA levels were decreased following RSV infection and the nuclear localization of the protein was decreased in infected cells compared to uninfected ones [107].

Translocation of NRF2 into the nucleus is integral to its effect on gene expression. For NRF2 to be imported into the nucleus and to express function requires a number of accompanying signals. During the nuclear transfer process, NRF2 associates with karyopherins known as importin α5 and importin β1 [108]. SARS-CoV-1 encodes several interferon antagonists that delay host cell recognition of infection, innate immune function, and interferon-stimulated gene expression. One such antagonist, ORF6 protein, does so by inhibiting nuclear import [109]. ORF6-mediated interferon antagonism is demonstrated to be mediated by the karyopherin chaperone protein. Proteins in the karyopherin-β family mediate the majority of macromolecular transport between the nucleus and the cytoplasm. Karyopherin β1 is essential for all nuclear import by karyopherin α proteins and depletion of this factor may dramatically reduce the transport of cargo by karyopherin-based transport. Thus, SARS-CoV1 prevents NRF2 translocation into the nucleus by inhibiting karyopherins. This results in inability to increase expression of genes involved in suppressing viral replication [110].

It has also been shown that coronavirus infection can inhibit the activity of numerous karyopherin-dependent host transcription factors including the vitamin D receptor (VDR) and hypoxia-inducible factor α2 [HIFα2]-Epas, p53 in human lung cells [109]. A cross-sectional study of 693 healthy subjects demonstrated that serum concentration of 25(OH)-vitamin D is positively associated with serum levels of GSH and cysteine as well as their respective oxidized disulfides [111]. It has been demonstrated that supplementation with exogenous vitamin D leads to increases in intracellular GCLC and glutathione reductase in a human monocyte cell line [112]. High-dose vitamin D_3_ supplementation in ventilated, critically ill patients decreases length of hospital stay [113]. Ironically, GSH plays a role in maintaining vitamin D regulatory genes; GSH deficiency hinders the expression of vitamin D-binding proteins and receptors [114,115]. Furthermore, supplementation with L-cystine, a precursor for GSH, increases levels of vitamin D and its binding proteins [114,116]. Similarly, an experiment conducted in transgenic mice demonstrated that administration of vitamin E can rescue formation of an embryonic inner cell mass in GPx4 -/- KO mice [66]. These data indicate that the dietary antioxidants vitamin D and vitamin E play a key regulatory role in the pathways of endogenous GSH synthesis. With respect to hypercytokinemia associated with SARS-CoV-2, we posit that supplementation with preformed liposomal GSH, as opposed vitamin D or GSH precursors, will provide a direct exposure to the biologically active molecule without exacerbating metabolic burden on potentially compromised pulmonary parenchyma in COVID-19 associated ARDS.

## 12. GSH and Pulmonary Disease

Individuals with ARDS appear to have a significant deficiency of GSH in the epithelial lining fluid of their respiratory tract. This plays a role in heightening oxidative burden, inciting inflammation and pulmonary edema [11]. The effect of oxidative stress in lung function impairment and decrease in antioxidant GSH has been shown in multiple studies. The GSH precursor NAC has been used as a treatment modality in numerous pulmonary ailments including chronic obstructive pulmonary disease (COPD) and chronic bronchitis. It has been shown to prevent COPD exacerbations at high dosages and chronic bronchitis flare- ups at regular doses [8]. A meta-analysis also demonstrated that individuals treated with NAC have significantly fewer exacerbations of COPD and chronic bronchitis [5]. NAC, when used as an adjunct treatment for idiopathic pulmonary fibrosis, exhibited promising results and is now part of the treatment guideline for idiopathic pulmonary fibrosis [29,30]. Another study showed that supplementation with GSH was able to stabilize the cytokine disparity in other viral diseases, which effectively mitigated inflammation patients with concomitant HIV and pulmonary TB [58].

Patients with ARDS have decreased GSH concentration in plasma and erythrocytes. In a randomized crossover study, patients who were given intravenous NAC, showed an increase in GSH levels. These individuals exhibited a clinical response to treatment with increased oxygen delivery, improved lung compliance, and resolution of pulmonary edema [117]. GSH depletion is also observed in alveolar epithelial tissue in patients with ARDS compared to normal subjects [118]. In a randomized controlled trial of patients with community acquired pneumonia, NAC used in conjunction with conventional therapy was shown to decrease inflammatory response in patients when compared to conventional therapy alone [119]. 

## 13. The Role of GSH in Ferroptosis

Ferroptosis is programmed cell death by iron and lipid dependent peroxidation [120]. It has been linked to ageusia and anosmia which are common early manifestations of COVID-19 illness [121,122,123]. Lipid peroxidation is carried by iron dependent lipoxygenases (LOXs). These enzymes lead to formation of lipid hydroperoxides from polyunsaturated fatty acids (PUFAs) and are expressed in immune cells. Increased levels of iron can enhance the iron dependent formation of lipid reactive oxygen species [124]. Ferroptosis is regulated by lipid repair enzymes, which include GSH and GPx4 [68]. GPX4 is a GSH-dependent enzyme that reduces lipid hydroperoxides (L-OOH) to lipid alcohols (L-OH). Toxic lipid peroxidation can also be reduced by import of cystine and GSH synthesis, as it boosts the function of GPx4 [125].

Given the data suggesting hyperferritinemia in patients with severe COVID-19, ferroptosis can be a significant contributor to tissue damage in COVID-19 illness. This combined with low endogenous levels of GSH and deficient GS and GCL action can result in a buildup of ROS and cell death. We hypothesize that administration of liposomal glutathione can boost intrinsic GSH levels, enhance GPx4 function and reduce tissue damage and cell death propagated by ferroptosis.

## 14. GSH and SARS-CoV-2

Evaluation of viruses in the family Coronaviridae reveals that considerable release of proinflammatory cytokines, including IL-2, IL-6, IL-7, IL-10, and TNF-α (Figure 1 and Figure 2), plays a crucial role in the pathogenesis of SARS-CoV-1 and MERS-CoV infections [16]. NOX4-derived ROS production has been shown to be modulated by angiotensin-converting enzyme 2 (ACE2) [126]. ACE2 is known to be the receptor for SARS-CoV-1 and is now also identified as the key receptor for the novel SARS-CoV-2 [127]. Glycosylation, a reaction that can be induced by hyperglycemia, of ACE2 is needed for the linkage of the virus to this cellular receptor [128]. 

Cytokine profiles of SARS-CoV-2 positive individuals show an elevation in IL-6, IL- 2, IL- 7, IL-10, and TNF-α. Similar to infections of SARS-CoV-1 and MERS-CoV, where cytokine release syndrome (CRS) was found to be the major cause of morbidity, in SARS-CoV-2 infection, elevated IL-6 correlates with severe respiratory failure [16]. Multiple studies have shown that COVID-19 progression to ARDS is initiated by hypercytokinemia, but increased serum IL-6 concentration specifically was found to correlate with ICU admission, progression to ARDS and mortality. Massive inflammatory reaction is observed when there is a dysregulated ratio of pro-inflammatory to anti-inflammatory cytokines (Figure 1 and Figure 2) [6,27].

GSH deficiency has been associated with a more severe clinical manifestation of coronavirus. One article with a small number of cases shows decreased plasma GSH is associated with more severe presentation of disease. Levels of GSH and ROS were measured in 4 patients with laboratory confirmed COVID-19. In the patients with higher baseline levels of GSH, there was an observed decrease in ROS, and these patients had a shorter course of illness. Decreased GSH was associated with increased ROS and more severe symptoms [35]. The role of GSH depletion in COVID-19 is a topic which warrants further analysis.

SARS-CoV-2 influences intracellular GSH levels by decreasing the function of intracellular NRF2, which plays an important role in protecting cells from oxidative damage by upregulating GSH production (Figure 1). In cells that are stressed, there is a release of NRF2 which then is escorted from the cytoplasm into the nucleus by an importin protein, karyopherins [108]. Coronavirus inhibits karyopherin-mediated nuclear import process thus decreasing the production of GSH [109].

Based on evidence provided from previous studies conducted by our laboratory, it can be reasonably concluded that administration of liposomal glutathione would be particularly beneficial in certain high-risk COVID-19 patients, such as those who are also suffering from type II diabetes mellitus or those who are co-infected with HIV or *M. tb*. Liposomal glutathione has been demonstrated to be able to augment Th1 cytokine response in patients suffering from HIV and *M. tb* infections [58]. Intracellular GSH concentration in erythrocytes is shown to be diminished in patients with type II diabetes [129]. Thus, we would expect these high-risk or immunocompromised populations to benefit in particular from liposomal GSH supplementation.

A case series in New York assessed the clinical courses of two COVID-19 patients diagnosed either by serology assay or chest CT findings. RT-PCR was not performed due to lack of available testing materials. Both patients presented in March 2020 with prodromal symptoms consistent with SARS-CoV-2 pneumonia, including dysgeusia and hyposmia. Each patient was placed on a trial of the GSH precursor NAC, α-lipoic acid, and 2 g doses of either oral or intravenous GSH. The patients started GSH for the first time approximately ten days after presentation, corresponding to the time of greatest severity of CT abnormalities in COVID-19 patients without ARDS [130]. The patients expressed that their dyspnea began to improve within 1 h of the GSH transfusion and continued to improve with each subsequent dose. With repeated doses, one patient was able to ambulate and perform activities of daily living without pre-syncopal episodes arising from dyspnea. Case 1 was treated with an array of medications including 2 g of intravenous GSH, which was observed to improve “his sense of well-being and quickly improved his dyspnea each time it was administered daily”. Case 2 was given oral liposomal GSH, which was “administered at 2000 mg PO due to superior efficacy” as reported by the patient. Although the sample size was small, the researchers demonstrated improvement in the patients following GSH administration and recommended that randomized controlled trials be conducted to evaluate the efficacy of GSH and its precursors in patients with COVID-19 pneumonia and ARDS [131]. 

New data regarding possible treatment avenues and pathogenesis of COVID-19 is emerging every day. As mentioned above, previous studies have shown that patients suffering from HIV/AIDS, tuberculosis and T2DM have relatively lower intrinsic levels of GSH and higher GSSG compared to their non-diseased counterparts [58,74,129]. It is our hypothesis that in the setting of COVID-19 and oxidative stress, patients with comorbidities may have compromised levels of GS and GCL, the enzymes involved in GSH synthesis For this reason, we suggest directly supplementing liposomal glutathione, instead of NAC as patients with deficient levels of GS and GCL will not be able to use NAC as substrate for GSH synthesis.

As mentioned earlier, GSH plays a major role in decreasing oxidative stress and boosts immune function. Evidence of this is presented in a study that looked at administration of oral liposomal GSH at 2 doses of 500 mg and 1000 mg per day in 12 healthy adults. These patients were followed at 1, 2, and 4 weeks with levels of GSH in whole blood, red blood cells, plasma cells and peripheral body mononuclear cells (PBMCs). The findings showed 100% increase in GSH levels in PBMCs. In addition, there was a statistically significant decrease in biomarkers used to track oxidative stress such as 8-isoprostane and ratio of GSSG to GSH. There was also an increase in NK cell cytotoxicity by 400% by 2 weeks This study though has a low statistical power due to a small sample size, shows the efficacy of liposomal GSH in increasing GSH levels in vivo and reducing oxidative stress [132]. No randomized controlled trials have been done to compare serum or intracellular GSH levels with administration of liposomal GSH vs. non-liposomal GSH. Liposomal formulations are used to deliver hydrophilic and lipophilic substances that may otherwise be degraded in the acidic environment of the stomach. It also prevents early inactivation, degradation, and dilution in the circulation [133,134]. Thus, we suggest supplying a bioavailable formulation of GSH in the form of reduced liposomal GSH as an adjunct in COVID-19 treatment.

## 15. Conclusions

COVID-19 represents a historic challenge to the fields of research, infectious disease, and international healthcare. The need for detailed analysis of its pathogenesis and clinical course is readily apparent. The unprecedented acuity of a rapidly spreading pandemic presents an opportunity to advance international collaboration in the scientific community. While vaccine trials remain ongoing, physicians have been compelled to apply various treatments with established efficacy in similar viral or bacterial illnesses that also lead to bilateral pneumonia and ARDS. Here we present the antioxidant GSH as a potential untapped avenue for further investigation as intervention for COVID-19. We propose to use a formulation that contains a predominately reduced form of glutathione in the formulation rather than oxidized. In a patient that is burdened with cytokine storm, the best thing for the immune system would be to supply it with reduced glutathione such that it is already able to supply reducing equivalents from its thiol group. Our work with HIV, TB, and other pulmonary or immunosuppressive illnesses demonstrates the value of GSH as an adjunct treatment for SARS-CoV-2 infection.
